# Thermophysics-Informed Phenomenological Framework for Molten Material Self-Organization in Laser Remelting-Based Surface Polishing: Conceptualization and Preliminary Analysis

**DOI:** 10.3390/mi17050528

**Published:** 2026-04-26

**Authors:** Evgueni Bordatchev

**Affiliations:** Mechanical and Materials Engineering, Western University, London, ON N6A 5B9, Canada; ebordatc@uwo.ca

**Keywords:** surface polishing, laser remelting, self-organization, chaos estimation, phase portrait, Lyapunov exponent, approximate entropy, Hurst exponent

## Abstract

The goal of laser polishing (LP) is to improve the surface quality of functional parts, components, and assemblies. LP is a complex nonlinear thermophysical process, in which laser radiation induces localized melting of a material with an initially rough surface topography. During LP, the thermodynamic state evolves dynamically due to transient melt flow, heat transfer, and rapid solidification within the laser–material interaction zone. A smooth surface is formed through the interplay between surface tension-driven flow, which promotes energy minimization, and nonequilibrium effects associated with melting and solidification. From the perspective of self-organization, LP can be interpreted as an open system driven by energy input, where complex material redistribution leads to the evolution of surface topography. In this work, the self-organization of molten material is analyzed using chaos-based descriptors, including the Lyapunov exponent, phase portrait, approximate entropy, and the Hurst exponent, calculated from measured surface topographies before and after laser polishing. The results show that LP acts as a spatial low-pass filter, reducing high-frequency surface components associated with micromilling marks, and exhibits a directional bias in material redistribution relative to the laser scanning direction. Among the evaluated descriptors, the Lyapunov and Hurst exponents demonstrate consistent behaviors, indicating their suitability as robust indicators of surface state in post-process analysis. For the investigated conditions (Inconel 718), a laser fluence of 158.3 mJ/cm^2^ provided the best-achieved surface quality, corresponding to an improvement in surface roughness (Ra) of approximately 70% and the lowest Lyapunov exponent of 1.966 and highest Hurst exponent of 0.859. This study demonstrates that chaos-based analysis of surface topography provides a phenomenological framework for assessing process stability and surface evolution, offering a basis for thermophysics-informed development of LP in applications such as mold and die manufacturing.

## 1. Introduction

Modern manufacturing continuously seeks new technologies to reduce costs and increase product quality and production efficiency. This mindset is especially true for the tooling industry, where a significant portion (e.g., up to 80% for optical tooling) of the total product cost is attributed to surface finishing operations, such as polishing and lapping. With respect to conventional finishing operations [1], only two novel finishing technologies exist that do not add or remove material: polishing by an electron beam [2] and polishing by a laser beam [3,4,5,6]. Both processes involve smoothing the original surface topography by material remelting. This study focuses on the latter process of surface polishing by laser remelting (SP-LRM), also known as laser polishing (LP).

LP is a complex, nonlinear thermodynamic process whereby laser irradiation heats and melts the surface of a material, while preferably avoiding, or at least minimizing, evaporation and ablation of the material. The laser–material interaction forms a pool of molten material that flows over a predetermined surface area. The two types of LP are defined by the surface scanning trajectories: (i) the laser beam is displaced over the material surface by a multi-axis galvanometric scanner, or (ii) the material is displaced with respect to the laser beam by a multi-axis electromechanical motion system in accordance with a pre-designed laser-spot trajectory. Each type involves simultaneous control of the laser power, scanning speed, and working distance [7]. The main advantages of LP are a high processing ratio (e.g., <0.1 s/cm^2^ [3]), computer numerical control, and significant surface quality improvement (up to 95% [6]).

The main disadvantages of LP are process complexity [8], a lack of understanding of how to optimize the process, and uncontrollable process instabilities that lead to nonuniform surface topographies [9,10]. Trial and error in experimental design are still required to find the optimal process parameters to obtain the desired and/or best-achievable surface quality. This technical challenge stems from two main factors: the significant complexity of the process thermodynamics and the large number of process parameters relating to the workpiece material, laser characteristics, laser–material interactions, laser-spot trajectory, initial surface geometry, and the geometry of the area to be polished.

The thermodynamics of LP depends critically on the applied laser fluence. Two principal thermodynamic regimes exist: (i) shallow LP, where the depth of the molten material is comparable to variations in the initial surface topography (e.g., <5 µm), and (ii) deep LP, where the peak-to-valley distance of the initial surface topography is negligible with respect to the depth of the molten material (e.g., >20 µm). This study analyzes how and where molten material flows and self-redistributes during LP in these two LP regimes. Building upon existing LP knowledge, surface smoothing by redistributing molten material is considered through the complex interplay between the order stemming from surface tension, which strives to minimize the surface energy and the thermodynamic chaos of rapid melting–solidification processes. The chaotic flow and self-organization of the molten material are analyzed by calculating chaos-characterization parameters (e.g., approximate entropy, the Lyapunov exponent, phase portraits, and the Hurst exponent) of the initial and laser-polished surface topographies. To be specific, each metric is considered as connected to LP thermophysics: the Lyapunov exponent estimates sensitivity to perturbations in the melt flow, the Hurst exponent shows persistence of surface formation mechanisms, and entropy evaluates the regularity of surface topography formation.

## 2. Thermodynamic Self-Organization and Thermophysics-Informed Dynamics of Laser Polishing: A Phenomenological Framework

### 2.1. Conceptual Thermodynamic/Self-Organization Understanding of Laser Polishing

Self-organization occurs in open, far-from-equilibrium systems that obey the second law globally while locally decreasing entropy [11,12]. Dynamic self-organizing processes do not reach a thermodynamic equilibrium because of so-called dissipative structures through which energy and/or matter flow. LP requires a fundamental understanding of how an initial surface topography (considered to be in an ordered state because it is a solid body) transitions into a disordered state (e.g., a liquid) during continuous laser remelting (LRM). Furthermore, LRM allows the material to transition back into a different ordered state via rapid solidification.

Several modeling studies [13,14] have considered the equilibrium thermodynamics of LP under conditions of reversibility and adherence to the first law of thermodynamics. Most such studies focus on heat transfer, temperature distribution, the location of the solid–liquid interface, and the shape and size of the molten pool but do not consider the temperature dependence of the physical-mechanical properties of the workpiece material. Few simulation studies have considered LP-induced surface topography modification by incorporating surface tension [15] or integrated multiple heat-transfer mechanisms such as conduction, convection, and radiation or the temperature dependence of the relevant material properties [16,17].

In self-organization theory [11,12], LP is considered a system “open to the flow of energy,” where fluctuation-induced instabilities produce nonrepeatable and nonuniform heat conduction, leading to abrupt transitions between nonrepeatable and repeatable melt-solidification phenomena. Based on the fundamentals of modern thermodynamics [11], one formulation of the second law of thermodynamics states that “every system tends to its most probable state.” What is the most probable state for the LP system in terms of technical function or purpose? It is a surface topography with the optimal or desired characteristics. In the ideal case, LP produces a flat surface with negligible topographical deviations from a mean value (i.e., standard deviation and variance are zero). By analogy with ice cream melting [18], LP performance can be illustrated by considering how the surface of a hockey rink is smoothed by melting a surface layer of solid ice, followed by freezing the resulting liquid layer to create a flat skating surface. After the surface is used (i.e., roughened), the rough ice surface can be remelted and resolidified again to regain its smooth state. LP is analogous—we create a smooth solid surface from an initially rough solid surface by melting a surface layer of the latter and allowing the liquid layer to resolidify. For LP, a moving laser beam melts a rough zone of the material surface, and the molten material, driven by thermodynamics, spreads to its most probable state (i.e., burying peaks and filling valleys), creating a locally smooth area. As the laser beam moves away, the molten material rapidly solidifies, self-organizing into a new surface topography. From a self-organization point of view, the surface topography constantly changes during LP, undergoing an organized-disorganized-organized cycle.

The phenomenological reasoning above argues that LP as a self-organizing process is a thermodynamically open system because it requires constant low-entropy input of energy from a laser to release the internally generated entropy through the output of heat (e.g., conduction, dissipation, and convection). This reasoning allows us to understand LP as an irreversible, dissipative process that may form a remelted surface with two principal qualities: (i) smoothed initial surface irregularities due to thermodynamically stable self-organization and (ii) irreversible degradation (e.g., roughening) of the initial topography due to thermodynamic instabilities in the laser–material interactions. Given that entropy measures irreversibility and dissipation, the thermodynamically stable self-organization is a relatively low-entropy (in the information-theoretic sense) regime of a smooth, homogeneous surface topography. In contrast, LP with maximum entropy characterizes the chaotic thermodynamic formation of surface nonuniformities. In practice, the study of the thermodynamics of LP can take two approaches: using control theory to study off-line the statistical transformation of the measured initial surface and the laser polished surface topographies as areal (spatial) data series [8,10,19] and the online analysis of thermographic images of the laser–material interaction zone [9,10,20,21]. This study takes the former approach by analyzing the self-organization of the molten material by calculating chaos-estimation parameters, such as the Lyapunov exponent, phase portrait, approximate entropy, and the Hurst exponent, from the measured topographies of the initial and laser polished surfaces.

### 2.2. Thermophysics-Informed Interpretation of Chaos Metrics in Laser Polishing

Laser polishing by laser remelting (LP/SP-LRM) is governed by strongly coupled thermophysical phenomena, including transient heat conduction, thermocapillary (Marangoni) convection, capillary-driven melt flow, and rapid solidification. These processes evolve within a highly localized and continuously moving melt pool, where small perturbations in energy input, material properties, or boundary conditions may lead to amplified responses in melt flow and, consequently, in the resulting surface topography. From a dynamical systems perspective, LP can therefore be interpreted as a nonlinear, dissipative, far-from-equilibrium process exhibiting sensitivity to initial and boundary conditions.

Within this framework, the measured surface topography is not merely a geometric outcome but represents a spatially resolved outcome of the underlying melt-pool dynamics. The transformation from the initial surface profile *h_ini_*(*x*) to the remelted profile *h_LP_*(*x*) reflects the integrated effect of local heat input, melt redistribution, and solidification kinetics along the laser trajectory. Consequently, statistical and chaos-related descriptors extracted from these profiles can be interpreted as indirect measures of the stability and organization of the governing thermophysical processes.

The Lyapunov exponent characterizes the sensitivity of the system to infinitesimal perturbations. In the context of LP, positive Lyapunov exponents indicate that small fluctuations in melt-pool geometry, temperature gradients, or flow velocity fields grow over time, leading to divergence in surface evolution trajectories. Physically, this behavior can be associated with instabilities in thermocapillary convection or fluctuations in the melt-pool boundary, which result in nonuniform material redistribution. Lower Lyapunov exponents, although still positive, suggest a more stable melt-pool regime in which perturbations are damped more effectively, leading to smoother and more predictable surface formation.

The approximate entropy provides a quantitative measure of the regularity and predictability of the surface profile. In thermophysical terms, a decrease in entropy from *h_ini_*(*x*) to *h_LP_*(*x*) indicates a transition from a surface dominated by deterministic machining marks and stochastic perturbations to a more uniform state governed by surface-tension-driven flow. This reflects the ability of the melt pool to act as a low-pass spatial filter, attenuating high-frequency components of the initial topography through viscous flow and capillary leveling. However, excessive entropy may reappear under unstable processing conditions, where competing mechanisms such as recoil pressure, localized overheating, or irregular solidification introduce additional complexity into the surface structure.

The Hurst exponent provides insight into the persistence or memory of the surface formation process. Values greater than 0.5 indicate persistent behavior, meaning that trends in surface evolution are maintained along the scanning direction. In LP, this persistence can be interpreted as a manifestation of coherent melt flow and stable energy input, where the redistribution of molten material follows a consistent directional pattern governed by the laser trajectory and thermocapillary forces. Conversely, values closer to 0.5 or below indicate increased stochasticity, suggesting that the melt-pool dynamics are dominated by random fluctuations rather than controlled flow, which leads to less predictable surface features.

Importantly, these chaos-based metrics should not be interpreted as purely abstract descriptors but as reduced-order representations of complex thermophysical interactions. Together, they capture complementary aspects of LP dynamics: the Lyapunov exponent reflects sensitivity to perturbations, approximate entropy characterizes structural regularity, and the Hurst exponent describes long-range correlations in surface evolution. Their combined analysis enables a more comprehensive interpretation of the transition between stable and unstable processing regimes.

From a process-engineering perspective, this thermophysics-informed interpretation establishes a foundation for linking measurable surface characteristics to underlying process stability. In particular, the identification of parameter ranges that minimize entropy, reduce Lyapunov divergence, and maximize persistence (Hurst exponent) provides a pathway toward defining quantitative stability criteria for LP. Such criteria are essential for the future development of data-driven and physics-informed control strategies, including real-time monitoring and digital twin-based optimization of laser remelting processes. It should be noted that the entropy measures used in this study refer to information entropy derived from surface topography and are employed as descriptors of spatial regularity and predictability. These measures should not be interpreted as thermodynamic entropy in the classical sense, but rather as phenomenological, reduced-order indicators that indirectly reflect the outcome of underlying thermophysical processes.

## 3. Analysis Methodology and Experimental Setup

The research methodology for this study was designed to explore the role of entropy-related dynamics in the self-organization of molten material during LP. LRM experiments were performed so that initial and laser-polished topographies were in close spatial proximity, which allowed their measured profiles to be statistically and functionally cross-related, ensuring reliable and repeatable estimation of chaos parameters. These were the main reasons for performing LRM experiments as single laser tracks over deterministically defined initial surface topography.

**Figure 1:**
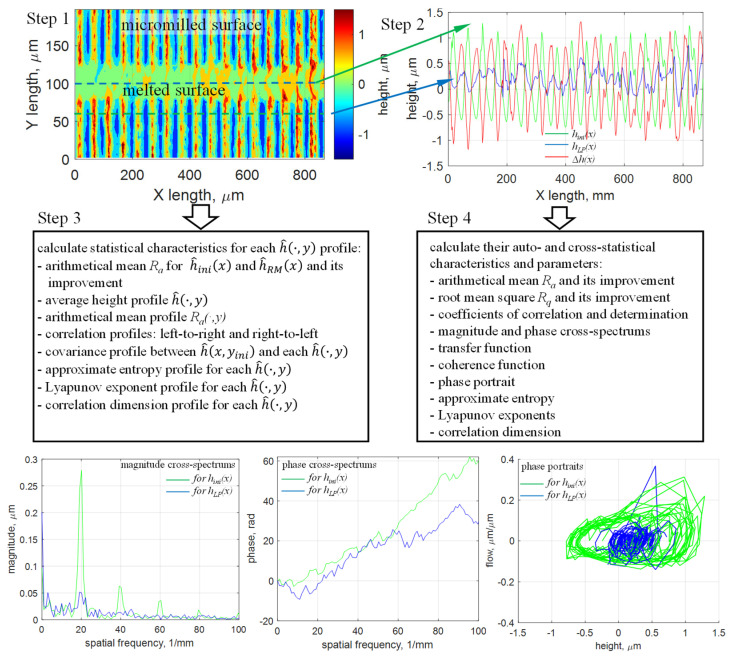
Methodology for analyzing self-organization during LP.

Figure 1 shows the methodology for analyzing self-organization during LP, which consists of the following steps:

1. Prepare the initial surface of an Inconel 718 superalloy sample by micromilling with a 0.6 mm ball end cutter and a step-over of 50 µm to obtain an average peak-to-valley amplitude of approximately 2 µm and an areal arithmetical mean, *S_a_* = 0.53 µm. The step-over of 50 µm was chosen because it is a widely accepted parameter used in the tool and die industry. This step outputs the topographies of initial and laser-polished surfaces, measured using an ultrahigh-precision profilometer (described later), as well as the initial information for detailed analysis of the self-organization process.
2. Extract at least two longitudinal profiles along the laser track with a minimum length of 800 µm (per the ISO 4288 standard [22]): one profile in the middle of the laser track as the laser-polished profile *h_LP_*(*x*) and another profile near the remelted material as the initial profile *h_ini_*(*x*). Calculate the difference between the laser-polished profile and the initial profile as ∆*h*(*x*) = *h_LP_*(*x*) − *h_ini_*(*x*). Calculating ∆*h*(*x*) in this way, as opposed to *h_ini_*(*x*) − *h_LP_*(*x*), assigns to ∆*h*(*x*) a physical meaning with respect to LP dynamics because it reveals the change in the initial surface profile at each X coordinate along the laser-spot trajectory. In addition, the offset of *h_ini_*(*x*) with respect to the remelted laser track is not critical because all profiles *h_ini_*(*x,y*) are highly cross-correlated (correlation coefficient of 0.98) within the micromilled area and have a consistent surface roughness *R_a_* = 0.509 ± 0.022 µm. Longitudinal profiles *h_ini_*(*x*), *h_LP_*(*x*), and ∆*h*(*x*) are the outputs of the second step of the analysis methodology.
3. Chaos-estimation parameters (e.g., the Lyapunov exponent, phase portrait, approximate entropy, the Hurst exponent) are calculated from eleven profiles of *h_ini_*(*x*), *h_LP_*(*x*), and ∆*h*(*x*) as quantitative measures for further analysis of self-organization.
4. In addition to estimating the chaos parameters, characteristics of the statistical transformation from *h_ini_*(*x*) to *h_LP_*(*x*) (e.g., correlation functions, power spectrums, coherence function, and others) can be calculated. From the perspective of LP dynamics, analyzing the phase spectrum is important because it shows how molten material is redistributed near the static riblets produced by micromilling.

**Figure 2:**
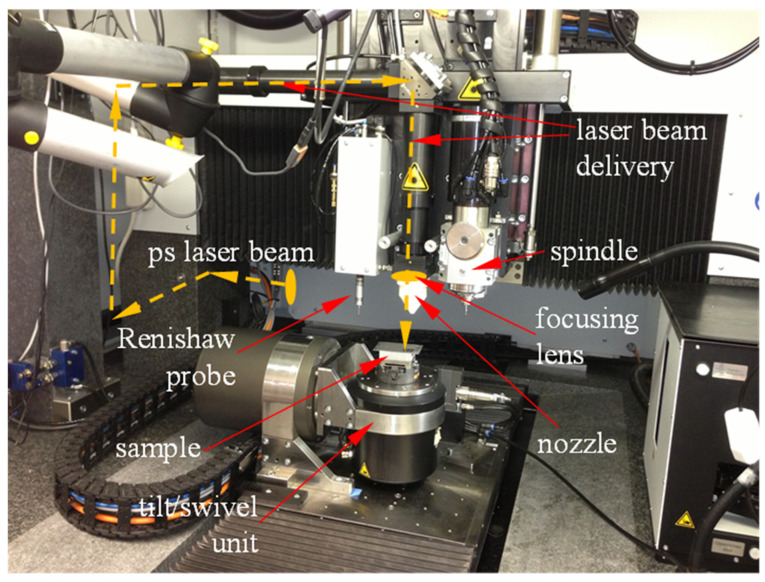
Multi-process, multi-axis high-precision micromachining system.

These comprehensive requirements for analyzing the self-organization methodology are not possible without the advanced capabilities [23] of the multi-process, multifunctional micromachining system MICROGANTRY nano5X (Kugler GmbH, Salem, Germany) used in this study (Figure 2). This system integrates several micromachining technologies and several measurement tools. The micromachining capabilities of this instrument include micromilling with a 125,000 rpm spindle, fly cutting with a 2000 rpm spindle, and micromachining with a picosecond laser. A Renishaw™ touch probe (Renishaw, West Dundee, CA, USA) with a measurement accuracy of ±500 nm is used to measure the workpiece geometry before and after machining and during alignment. The system is also equipped with a Blum™ tool-setting sensor (Blum, Lowesville, NC, USA) for measuring the cutting tool geometry (e.g., diameter and length) with a measurement repeatability of 2σ = 100 nm. The motion stages of this system ride on air bearings with a position resolution of 10 nm and a positioning accuracy of ±250 nm in the X-Y plane and ±500 nm in the Z direction. Straightness is within ±800 nm per 100 mm translation for all linear axes.

The system is also equipped with a Duetto picosecond laser (Time-Bandwidth Products, Inc., Switzerland) with a maximum average power of 12 W, a pulse width of 10.5 ps, a repetition rate up to 8.2 MHz, and an operating wavelength of 1064 nm. A workpiece was mechanically mounted on the A/C-axis tilt/swivel motion stage and aligned along the X and Z axes with ±250 nm accuracy using the Renishaw™ touch probe. This alignment procedure was applied in each experiment and allowed the workpiece to be removed and replaced back with high repeatability, which was needed to measure the surface topography before and after LP. Melting occurred in the focal range of 0.8–1.2 mm, where the laser fluence was 0.13–0.18 J/cm^2^ [24].

For the preliminary analysis of molten material self-organization presented below, three LRM experiments were performed with a laser fluence of 131.4, 143.9, 158.3, 166.3, and 184.3 mJ/cm^2^ and a scanning speed of 20 mm/s. Each LRM experiment produced two remelted regions: one 4.5 mm × 4.5 mm region and a single 4.5 mm long remelted line (the “laser track”). The surface topographies of the remelted lines were measured using an optical interferometer WYKO NT1100 (Veeco, Ltd., USA) with a vertical resolution of 0.1 nm and an X-Y grid size of 1.941 µm. This study analyzed only single remelted lines.

The number of experiments conducted in this study was intentionally limited to a small, well-controlled set of processing conditions. The primary objective of this work is not to establish a comprehensive parametric optimization of the laser polishing process, but rather to develop and demonstrate a thermophysics-informed phenomenological framework for interpreting molten material self-organization. To ensure the validity of this framework, experiments were designed under tightly controlled conditions, including a fixed scanning speed and a narrow range of laser fluences, allowing the isolation of fundamental thermophysical mechanisms governing melt-pool dynamics and surface evolution. Therefore, it should be noted that the analyzed surface topography represents a post-process outcome of coupled thermophysical phenomena, and the derived metrics describe the resulting structure rather than directly measuring transient melt-pool dynamics.

This reduced experimental design enables a clear interpretation of the relationship between process conditions, melt-pool behavior, and the resulting chaos-based descriptors, without the confounding effects introduced by a high-dimensional parameter space. Such an approach is consistent with first-stage exploratory and hypothesis-driven investigations, where the emphasis is placed on establishing physically meaningful relationships rather than exhaustive empirical mapping. Future work will extend this framework to a broader range of process parameters and operating regimes, including variations in scanning speed, multi-track interactions, and different materials.

## 4. Analysis of Molten Material Self-Organization

This study used two approaches for studying the self-organization of molten material redistribution during surface polishing by laser remelting: (i) the comparative analysis of the longitudinal profiles before (micromilled) and after (remelted) LP and (ii) the transitional evolution of three-dimensional (3D) topographies from the initial (micromilled) surface into the remelted area. There is a significant difference between these two approaches: the one-dimensional profile-based approach takes two longitudinal surface cross sections, one at the center of the remelted line and one near the laser track. In the 3D topography-based approach, the entire surface including the micromilled and remelted areas is analyzed by calculating the self-organization parameters for each longitudinal profile along the laser-track direction.

### 4.1. Comparative Analysis of Micromilled and Laser Remelted (LRM) Profiles

As a first step in understanding the self-organization of molten material under laser irradiation, consider a transformation from one equilibrium system represented by a micromilled surface profile into another equilibrium system represented by a remelted surface profile. In this scenario, the laser beam irradiates the solid body, which is an open system with a regular surface profile produced by micromilling. As a result, self-organization occurs during concurrent melting–solidification phenomena, so the initial solid profile loses its regularity and equilibrium due to the irreversible formations and phase transformations of dissipative structures during laser–material interactions. The dynamically changing thermal balance of the heat transport iteratively changes the thermal and thermocapillary flows and surface tension forces, thereby changing the shape and dimensions of the melt pool and solidified material. The ideal goal of LP is to obtain a flat solidified topography through the optimal combination of self-organization with LRM. Therefore, it was assumed that any deviation of the remelted profile from a flat line is an undesirable deviation from the target surface state that can be interpreted as an indicator of process instability. In a simplified interpretation, stable LRM tends toward forming a deterministically defined topography (e.g., a straight-line profile).

**Figure 3:**
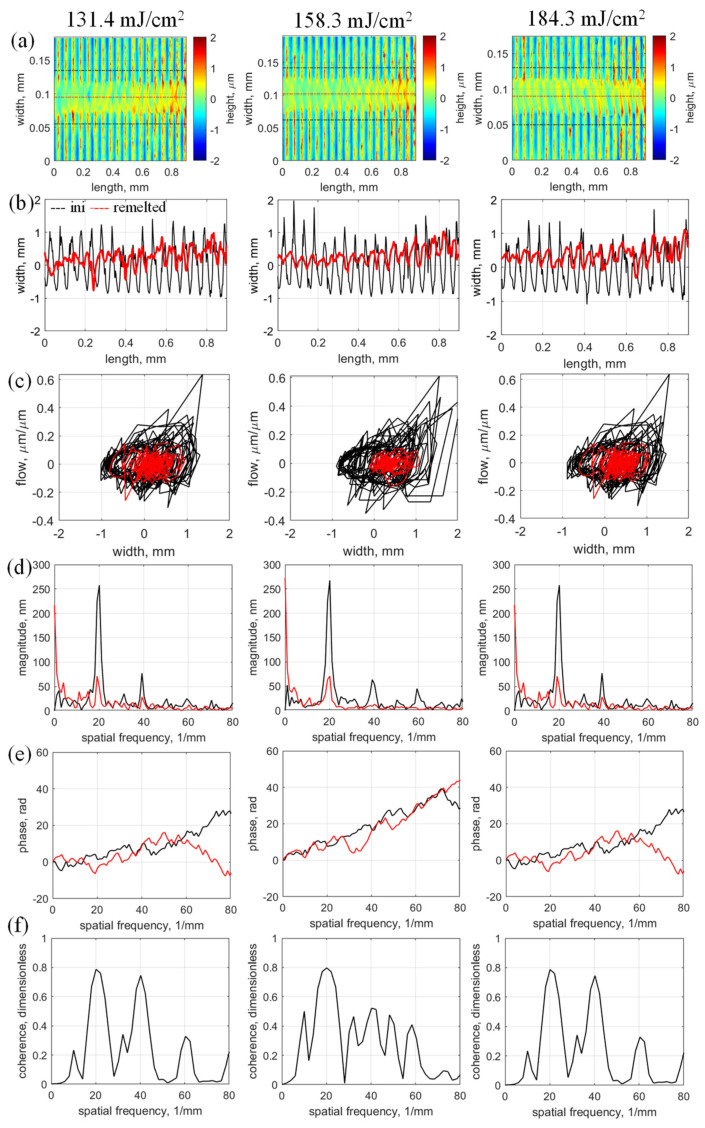
Experimental data and corresponding analysis results obtained with different laser fluences: (**a**) measured surface topographies, (**b**) central profiles, (**c**) phase portraits of central profiles, (**d**) amplitude-frequency characteristics, (**e**) phase-frequency characteristics, and (**f**) coherence functions between central profiles.

Taking this approach, samples with surface topographies *h*(*x,y*) obtained under different laser fluences (Figure 3a) were used to investigate the LRM performance. Two central profiles, initial *h_ini_*(*x*) (micromilled) and laser-polished (laser remelted) *h_LP_*(*x*), were extracted from each *h*(*x,y*), as shown in Figure 3b. Three main observations are revealed from the visual comparison of these profiles: the peaks of the micromilled profile decrease significantly during LRM, although the initial periodicity persists because the molten material does not evenly fill the micromilled tracks. In addition, new peaks in *h_LP_*(*x*) shift in the direction opposite to the laser-scanning velocity. These observations are supported by the surface roughness and chaotic behavior characteristics presented in Table 1 and by the calculated statistical characteristics of *h_ini_*(*x*) and *h_LP_*(*x*) central profiles: phase portraits (Figure 3c), amplitude-frequency characteristics (Figure 3d), and phase-frequency characteristics (Figure 3e), and coherence functions between *h_ini_*(*x*) and *h_LP_*(*x*) (Figure 3f).

Surface quality, estimated by the arithmetical mean *R_a_* and root mean square *R_q_*, increases significantly with respect to the original surface quality for all laser fluencies, with the maximal (70.0%) reduction of *R_a_* occurring 529.6 nm to 159.1 nm at 158.3 mJ/cm^2^ fluence. *R_q_* varies in the same manner, representing the alterability of the surface profile. A maximum negative correlation coefficient of −0.205 occurs at 166.3 mJ/cm^2^ fluence, confirming the above-mentioned visual observation of the profile resolidifying in the direction opposite the laser-scanning velocity.

The next step of the analysis involves calculating the approximate entropy [25] for *h_ini_*(*x*) and *h_LP_*(*x*) as a measure of the system multimodality and complexity. Regular, predictable behavior is indicated by low entropy, whereas chaotic fluctuations result in high entropy. This interpretation of the approximate entropy can be applied to the optimization of the LP process. As seen from Table 1, while approximate entropy is quite consistent for all initial profiles (0.866, 0.828, 0.834, 0.892, 0.838), an increase in the laser fluence (131.4, 143.9, 158.3, 166.3, 184.3 mJ/cm^2^) decreases gradually the approximate entropy to 0.890, 0.835, 0.786, 0.733, 0.722, respectively. This observation is consistent with the expected behavior, as LP improves the surface quality, so the relatively regular (i.e., periodical) *h_ini_*(*x*) micromilled profile changes through self-organization to the more regular and more predictable *h_LP_*(*x*) remelted profile (i.e., a line in the ideal case).

The system dynamics represented by the profiles *h_ini_*(*x*) and *h_LP_*(*x*) can be qualitatively described by phase portraits (Figure 3c). As expected, the phase portraits of the *h_ini_*(*x*) profiles are very similar, with all having significant variations in the width and flow coordinates, typically within a phase area of ±1 µm × ±0.4 µm. LP “squeezes” the phase portraits; for the best-achieved surface quality achieved with 158.3 mJ/cm^2^, it covers only a phase area of ±0.5 µm × ±0.1 µm. The level of chaos in phase portraits was estimated by using the Lyapunov exponent as a measure of instability and infinitesimally perturbing each phase trajectory [26]. For all experiments, the Lyapunov exponents of the profiles *h_ini_*(*x*) and *h_LP_*(*x*) are positive, which statistically indicates the presence of chaos. However, the Lyapunov exponents of the profiles *h_LP_*(*x*) all exceed those for *h_ini_*(*x*), and the lowest Lyapunov exponent of 1.966 for all *h_LP_*(*x*) profiles corresponds to the best-achieved surface quality (obtained at 158.3 mJ/cm^2^). These results suggest potential for future development of monitoring or control strategies, particularly when combined with in-situ measurement approaches.

Figure 3d and Figure 3e show the amplitude- and phase-frequency characteristics, respectively, and reveal how the frequency components of *h_ini_*(*x*) and *h_LP_*(*x*) change during LRM self-organization. One such component dominates the others because it involves a periodicity with a micromilling step-over of 50 µm (a spatial frequency of 20 mm^−1^). Laser remelting significantly reduces the amplitude of this component; for example, the amplitude decreases from 293.2 nm for *h_ini_*(*x*) to 70.6 nm for *h_LP_*(*x*) (75.9% reduction) at a laser fluence of 158.3 mJ/cm^2^ per pulse. The spatial direction of the material redistribution is more clearly demonstrated by the phase-frequency characteristics, where a negative phase shift occurs at a frequency of 20 mm^−1^ (e.g., −9.7 rad for 131.4 mJ/cm^2^). This supports the visual observations from Figure 3b, where 20 mm^−1^ peaks shift in the direction opposite the laser-scanning velocity. Note that the 20 mm^−1^ component reaches 0.8 in all coherence functions (Figure 3f), which means that a linear dynamics model can be applied to this profile component to describe the transformation of *_ini_*(*x*) to *h_LP_*(*x*) for further development of LRM optimization and control.

In a common case, the profiles *h_ini_*(*x*) and *h_LP_*(*x*) are the areal series where each profile can be considered a combination of deterministic and random components. For example, *h_ini_*(*x*) contains a periodic component from a micromilling process and a random component from the cutting-force dynamics and plastic deformations of the material. These components affect self-organization during LRM and dynamically transform it into deterministic (e.g., linear) and random (e.g., profile deviations) components of the *h_LP_*(*x*) profile. This result explains why the qualitative and especially quantitative estimations of the deterministic and random components in *h_ini_*(*x*) and *h_LP_*(*x*) are critical for analyzing self-organization during LRM. The Hurst exponent [27] was used to estimate the ratio between deterministic and stochastic (random) components. The criteria for such estimation are based on the meaning of the Hurst exponent: (i) it equals 0.5 if the data series represents ideal white noise; (ii) for a Hurst exponent in the range [0.5, 1.0], the data series has a clear trend, with relatively low noise (i.e., persistent behavior); and (iii) antipersistent behavior occurs for a Hurst exponent in the range [0.0, 0.5], indicating that the data take an opposite value around a mean value [27]. In performed experiments (Table 1), the Hurst exponent for *h_ini_*(*x*) is always slightly above 0.5 level and less than that for all *h_LP_*(*x*) profiles, which are consistently above 0.7 level. This result means that areal series of *h_ini_*(*x*) is statistically closer to noise than the areal series of *h_LP_*(*x*). In contrast, the areal series of *h_LP_*(*x*) has fewer random deviations because they are smoothed LRM, and the objective of LP is to produce clear evidence of a linear trend in the form of a straight-line profile. The maximum Hurst exponent of 0.859 occurs at 158.3 mJ/cm^2^ for the best-achieved surface quality, which most strongly corresponds with a flat polished profile. These observations show that the Hurst exponent suggests potential for process characterization and optimization and may support future monitoring strategies when integrated with real-time sensing approaches.

**Figure 4:**
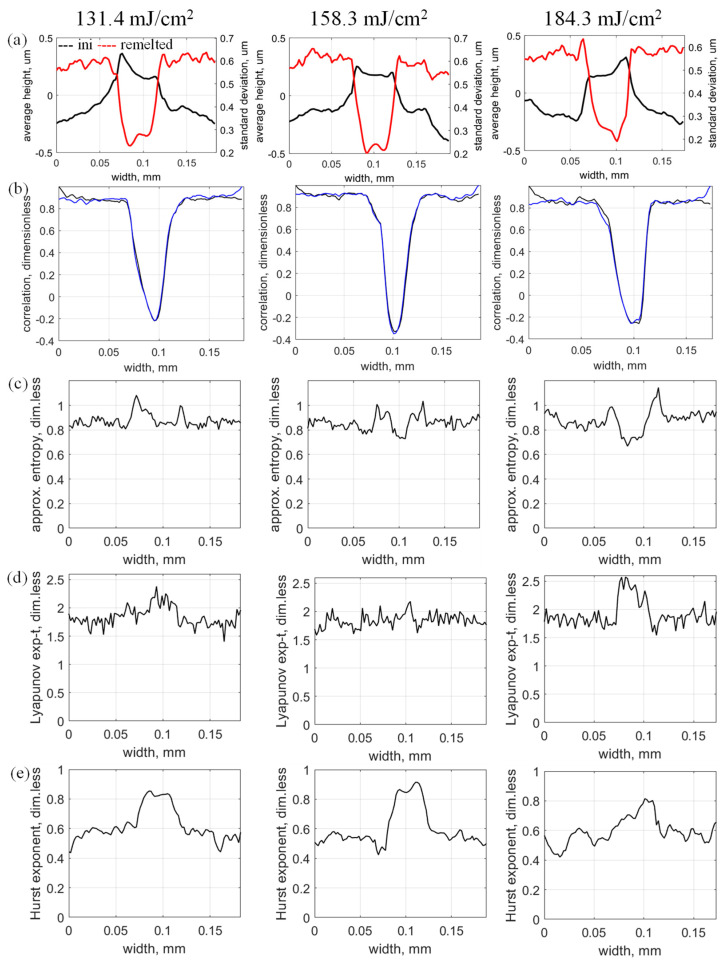
Experimental data and corresponding longitudinally calculated parameters of self-organization for different laser fluences: (**a**) averaged height and standard deviations of initial and remelted topographies, (**b**) correlation between longitudinal profiles, (**c**) approximate entropies, (**d**) Lyapunov exponents, and (**e**) Hurst exponents.

As per the analysis in Section 4.1, arbitrary longitudinal profiles *h_ini_*(*x*) and *h_LP_*(*x*) were selected within the micromilled sample area and in the middle of the LRM track. Therefore, *h_ini_*(*x*) does not suddenly transform into *h_LP_*(*x*) because the laser beam has a finite diameter, so the laser track has a finite width. In addition, given that the laser beam has a specific power distribution (e.g., Gaussian), the micromilled area gradually transforms into the LRM track and back again into the micromilled area (Figure 3a). In our experiments, the laser-track width was about 53 µm, so self-organization occurred during LRM within this width and in the corresponding melt pool. Thus, the next step in the analysis of the self-organization focuses on the distribution and evolution of the topography and chaos-estimation parameters, such as average height (Figure 4a, black line), standard deviation (Figure 4a, red line), correlation coefficient (Figure 4b), approximate entropy (Figure 4c), Lyapunov exponent (Figure 4d), and Hurst exponent (Figure 4e). Each of these plots was obtained by calculating the given parameter for each longitudinal surface profile across the scanned area.

The evolution of the surface topography amplitude was analyzed based on the average height (Figure 4a, black line). As expected from previous studies, a linear bulge of the remelted material forms, producing a riblet on the surface. The bulge width corresponds to the width of the melt pool. The variations in the bulge height are estimated by the standard deviation (Figure 4a, red line). As the surface topography becomes smoother under LRM self-organization, the standard deviation decreases within the boundaries of the LRM track. This transition from the micromilled area to the LRM area is not sudden and depends on the laser power distribution. The transition region may shrink if the Gaussian distribution becomes a top-hat distribution. The standard deviation also reflects topography variations around the desired flat profile of the polished surface.

Covariance cross sections (Figure 4b) show the repeatability of the profiles from left to right (black line) and from right to left (blue line). In both cases, the correlation coefficient remains fairly constant for *h_ini_*(*x*) profiles because it is repeatedly produced by micromilling. During LRM, the *h_ini_*(*x*) profiles change significantly, and the correlation coefficient for the *h_LP_*(*x*) profiles becomes negative (e.g., −0.203 for 158.3 mJ/cm^2^ laser fluence). This result confirms that periodicity remains in the *h_LP_*(*x*) profiles inherited from *h_ini_*(*x*), accompanied by a phase shift.

### 4.2. Analysis of the Transitional Evolution of 3D Topography

The relatively uniform cross-sectional plots of the approximate entropy (Figure 4c) and Lyapunov exponent (Figure 4d) do not provide evidence of a transitional evolution of 3D topography. Therefore, these characteristics may not be highly reliable for estimating self-organization during LRM. In contrast, the Hurst exponent is a more informative parameter for this purpose because its cross-sectional plot produces a well-defined pattern for the laser–material interaction zone (i.e., melt pool). The Hurst exponents for the *h_ini_*(*x*) and *h_LP_*(*x*) profiles directly correlate with improved surface quality, as demonstrated for a laser fluence of 158.3 mJ/cm^2^.

## 5. Summary and Conclusions

This study presents a thermophysics-informed analysis of molten material self-organization during surface polishing by laser remelting and demonstrates the applicability of chaos-based descriptors for characterizing process stability and surface topography evolution. In the performed LP experiments, initial surface topographies were generated by micromilling and subsequently modified by laser remelting using picosecond laser micromachining.

The transformation of the initial micromilled surface was analyzed systematically using profile- and areal-based parameters, including arithmetical mean and root mean square roughness, correlation characteristics, phase portraits, amplitude–frequency and phase–frequency responses, coherence function, approximate entropy, and Lyapunov and Hurst exponents.

The results show that laser polishing acts as a spatial low-pass filter, effectively reducing high-frequency components associated with micromilling marks and leading to significant smoothing of the surface topography. This confirms the capability of LP to attenuate periodic structures induced by prior machining processes.

In addition, the analysis reveals a directional bias in material redistribution, where newly formed surface features exhibit a systematic shift opposite to the laser scanning direction. This observation highlights the influence of process kinematics on surface formation and may have implications for tool-path planning in complex geometries.

Among the evaluated descriptors, the Hurst exponent demonstrates consistent behavior across processing conditions, indicating its suitability as a robust indicator of surface state and structural organization in post-process analysis.

For the investigated conditions (Inconel 718, fixed scanning speed), a laser fluence of 158.3 mJ/cm^2^ provided the best-achieved surface quality, corresponding to an improvement in surface roughness (Ra) of approximately 70% and the lowest Lyapunov exponent of 1.966 and highest Hurst exponent of 0.859 within the studied parameter range.

Overall, the proposed framework establishes a phenomenological foundation for linking measurable surface characteristics with process stability and provides a basis for further development of thermophysics-informed approaches to laser polishing.

These findings support the use of laser polishing as a controlled post-processing technique for mitigating machining-induced surface features and for guiding process parameter selection based on a thermophysically informed interpretation of surface evolution.

## Figures and Tables

**Table 1:** Parameters of surface roughness and chaotic behavior characteristics.

	ini	LRM	ini	LRM	ini	LRM	ini	LRM	ini	LRM
fluence, mJ/cm^2^	131.4		143.9		158.3		166.3		184.3	
*R_a_* of ini/LRM profiles, nm	511.6	176.1	505.0	164.5	529.6	159.1	515.6	176.3	484.0	188.2
*R_a_* improvement, %	65.6		67.4		70.0		65.8		61.1	
*R_q_* of ini/LRM profiles, nm	594.6	229.1	604.8	197.9	617.3	197.2	615.6	218.4	561.3	234.5
correlation coef. bw ini/LRM profiles, dimensionless	−0.143		−0.052		−0.203		−0.205		0.010	
coef. of determination bw ini/LRM profiles, dimensionless	0.021		0.003		0.041		0.042		0.000	
approximate entropy of ini/LRM profiles, dimensionless	0.866	0.890	0.828	0.835	0.834	0.786	0.892	0.733	0.838	0.722
Lyapunov exponent of ini/LRM profiles, dimensionless	1.777	2.097	1.828	2.133	1.788	1.966	1.805	2.081	1.794	2.366
correlation dimension of ini/LRM profiles, dimensionless	1.962	1.868	1.989	1.916	1.898	1.933	1.933	1.848	1.931	1.997
Hurst exponent of ini/LRM profiles, dimensionless	0.582	0.832	0.561	0.771	0.541	0.859	0.594	0.836	0.587	0.703

## Data Availability

The original contributions presented in this study are included in the article. Further inquiries can be directed to the author.

## Abbreviations

The following abbreviations are used in this manuscript:

3D	Three-dimensional
LP	Laser polishing
SP	Surface polishing
LRM	Laser remelting(ed)
SP-LRM	Laser polishing by laser remelting
